# Perceptually unidentifiable stimuli influence cortical processing and behavioral performance

**DOI:** 10.1038/s41467-020-19848-w

**Published:** 2020-11-30

**Authors:** Sorin A. Pojoga, Natasha Kharas, Valentin Dragoi

**Affiliations:** Department of Neurobiology and Anatomy, McGovern Medical School, Univ. of Texas-Houston, Houston, TX 77030 USA

**Keywords:** Sensory processing, Visual system

## Abstract

Our daily behavior is dynamically influenced by conscious and unconscious processes. Although the neural bases of conscious experience have been extensively investigated over the past several decades, how unconscious information impacts neural circuitry and behavior remains unknown. Here, we recorded populations of neurons in macaque primary visual cortex (V1) to find that perceptually unidentifiable stimuli repeatedly presented in the absence of awareness are encoded by neural populations in a way that facilitates their future processing in the context of a behavioral task. Such exposure increases stimulus sensitivity and information encoded in cell populations, even though animals are unaware of stimulus identity. This phenomenon is consistent with a Hebbian mechanism underlying an increase in functional connectivity specifically for the neurons activated by subthreshold stimuli. This form of unsupervised adaptation may constitute a vestigial pre-attention system using the mere frequency of stimulus occurrence to change stimulus representations even when sensory inputs are perceptually invisible.

## Abstract (web summary)

Perceptually unidentifiable stimuli are encoded in the visual cortex, such as to facilitate their future processing in a task context. This form of priming may constitute a pre-attention mechanism using the mere frequency of stimulus occurrence to change stimulus representations, even when sensory inputs are perceptually invisible.

## Introduction

The relationship between brain activity and behavior has been traditionally investigated by measuring the responses of neurons to stimuli presented above the detectability threshold. However, whether stimuli below the limit of awareness can influence brain responses to bias behavioral decisions is unknown. For instance, when an image is presented in close spatiotemporal proximity with other stimuli it becomes invisible, a perceptual phenomenon called masking. Perceptual studies have revealed that masked stimuli presented below the visibility threshold do not elicit conscious perception as they cannot be recognized or even guessed above chance on a force-choice test^1–6^. Nonetheless, they can facilitate the perceptual processing of the same stimuli in a subsequent behavioral task^1–5,7–11^. Although this phenomenon has been known for decades, how sensory information is encoded in the brain in the absence of awareness to influence subsequent sensory processing across neural circuits has remained a mystery. Specifically, do weak, subliminal stimuli unconsciously activate neuronal networks involved in perception such as to change sensory representations when stimuli are subsequently presented above the detectability threshold?

Previous psychophysics experiments have suggested that subliminal visual priming may be mediated by higher brain areas, possibly at or beyond the anterior part of the inferior temporal cortex^11^. Furthermore, functional imaging studies in humans using masked words have revealed that subliminal stimuli activate higher-order fusiform and precentral brain areas^6^. In addition, functional MRI recordings have revealed that perceptually invisible information can be briefly maintained at the higher processing stages of visual perception^12^, but not in early cortical areas. In light of this evidence, it has been proposed that the locus of subliminal priming is at an intermediate stage in the ventral cortical pathway for shape recognition, not in early visual cortex^1,6,11^. Previous electrophysiological experiments in monkey V1^13^ have reported that firing rates of individual cells are strongly reduced when forward and backward masks are combined, but whether and how exposure to such masked stimuli alters stimulus representation and subsequent perceptual performance has not been investigated. We address these issues for the first time by recording neural population activity in macaque primary visual cortex (V1) using multiple electrodes to examine whether perceptually unidentifiable stimuli influence the coding of information in visual cortex and perceptual performance. We demonstrate that exposure to unidentifiable stimuli presented below the threshold of perception changes stimulus representation as early as primary visual cortex to influence behavioral performance specifically for the stimuli being exposed.

## Results

### Designing perceptually unidentifiable stimuli

**Fig. 1:**
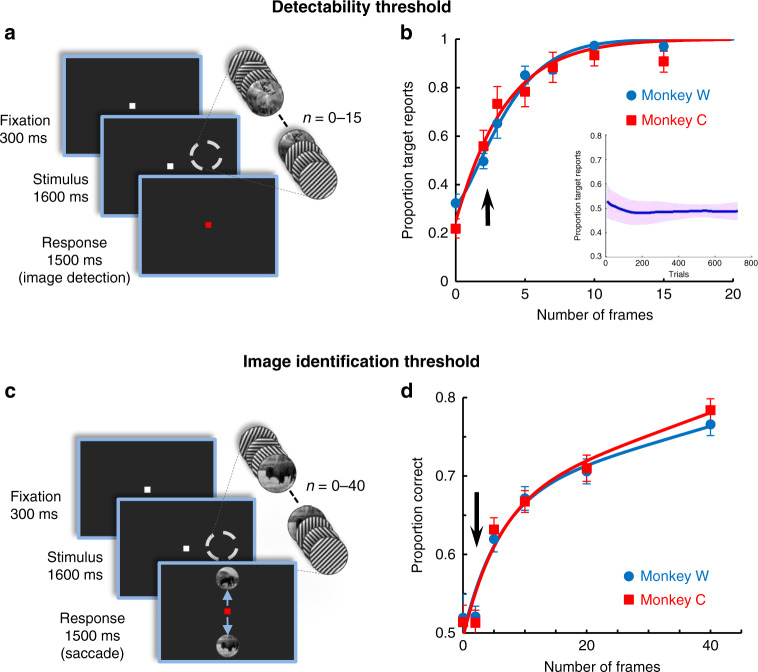
Behavioral experiments. **a** Cartoon describing the image detection task—monkeys were trained to report whether a natural image is embedded into a 60-Hz movie stimulus (50% of trials did not contain the image). The image was flashed for 2–15 consecutive frames inserted at random times with respect to movie onset (total movie length: 96 frames, 1600-ms). **b** Psychometric curves for the image detection task represented as a function of the number of consecutive image frames. Detection threshold (calculated at *d’* = 1) was 2.89 frames for monkey C and 3.28 frames for monkey W (*n* = 16, 6). Inset shows the average detection performance for the two-frame image condition as a function of trials (300 trials moving average, 10 ms step). **c** Cartoon describing the image identification task—one of two images, or no image in control trials, was randomly inserted into a movie stimulus (0–40 consecutive frames, same movie parameters as in **a**). Monkeys were required to identify the image by making a saccade toward one of the two scenes displayed after stimulus presentation. **d** Results of the image identification experiment represented as proportion correct saccades for stimuli containing between 0 and 40 image frames. Image identification performance for the two-frame stimuli was not different from chance (no image present in the movie; *P* = 1, *P* = 0.91, Wilcoxon sign-rank test, *n* = 20, 12). Error bars in all panels represent sem.

Natural scenes were rendered indistinguishable by embedding them into a 1600-ms movie, consisting of random oriented gratings flashed at 60 Hz^14–16^. To determine the exposure time for which a masked image becomes imperceptible, we first calculated the detectability threshold by varying the number of successive image frames across trials while monkeys reported whether the masked image was present or not in the movie (Fig. 1a—see Methods). Detection thresholds (Fig. 1b)—2.89 frames (48.23 ms) for monkey C, and 3.68 frames (61.33 ms) for monkey W—indicate that presenting two consecutive image frames (33.33 ms) embedded within the movie ensures that they fall below the detectability threshold (the corrected detectability performance after subtracting the false alarm rate is 25% and 41%, respectively, for the two monkeys). Importantly, the perceptual reports associated with two-frame images were stable and consistently below the detectability threshold during the time course of the experiment (Fig. 1b—inset). Notably, visual priming experiments in humans using subliminal stimuli reported relatively similar values for the detection threshold of masked targets^11,16–19^.

Even though the presentation of a two-frame image falls below the detectability threshold, we further examined if the animal is aware of image content, i.e., whether this stimulus can be identified in a forced-choice task (Fig. 1c). This task is particularly important in the context of our main study, which focuses on the conscious perception (identification) of stimuli by measuring the degree of discrimination between two images (see below) rather than the presence or absence of an image. Thus, we used 1600-ms movie strips (similar to those in Fig. 1a) containing a two-frame natural scene that could vary between two distinct images, image 1 and image 2 on a trial basis. The movie was presented during fixation and was followed by a forced-choice saccade task in which monkeys reported image identity (see Methods). Additionally, we varied the number of consecutive image frames to test the relationship between two image frames or >5 image frames stimuli and image identification performance. As expected, the image identification performance was significantly above chance for masked images lasting five frames or more (*P* < 0.001, Wilcoxon sign-rank test, *n* = 20,12). However, for two-frame images, performance was not significantly different from chance or false alarm rate (Fig. 1d, *P* = 0.9, Wilcoxon sign-rank test).

Furthermore, we performed additional psychophysical experiments in humans confirming that image content was not identified when two-frame images, identical to those used in the monkey experiments, were used in forced-choice category identification sessions (see Methods and Supplementary Fig. 1). Specifically, subjects were neither able to recognize subthreshold images presented for two frames, i.e., whether the image was a mammal or a bird (*n* = 9 subjects, proportion correct responses = 0.49, *P* = 0.5781, Wilcoxon sign-rank test), nor identify subthreshold images from a set of five images (*n* = 15 subjects, proportion correct responses = 0.21, *P* = 0.8374, Wilcoxon sign-rank test). Therefore, in all these tasks, subjects (monkeys and humans) are unable to perceive the image content as their identification performance was below threshold and not different from the false alarm rate (*d’* = 0). These experiments demonstrate that presenting two consecutive image frames (33.3 ms) embedded within the movie ensures that image content is not consciously perceived.

### Population coding of subthreshold stimuli

**Fig. 2:**
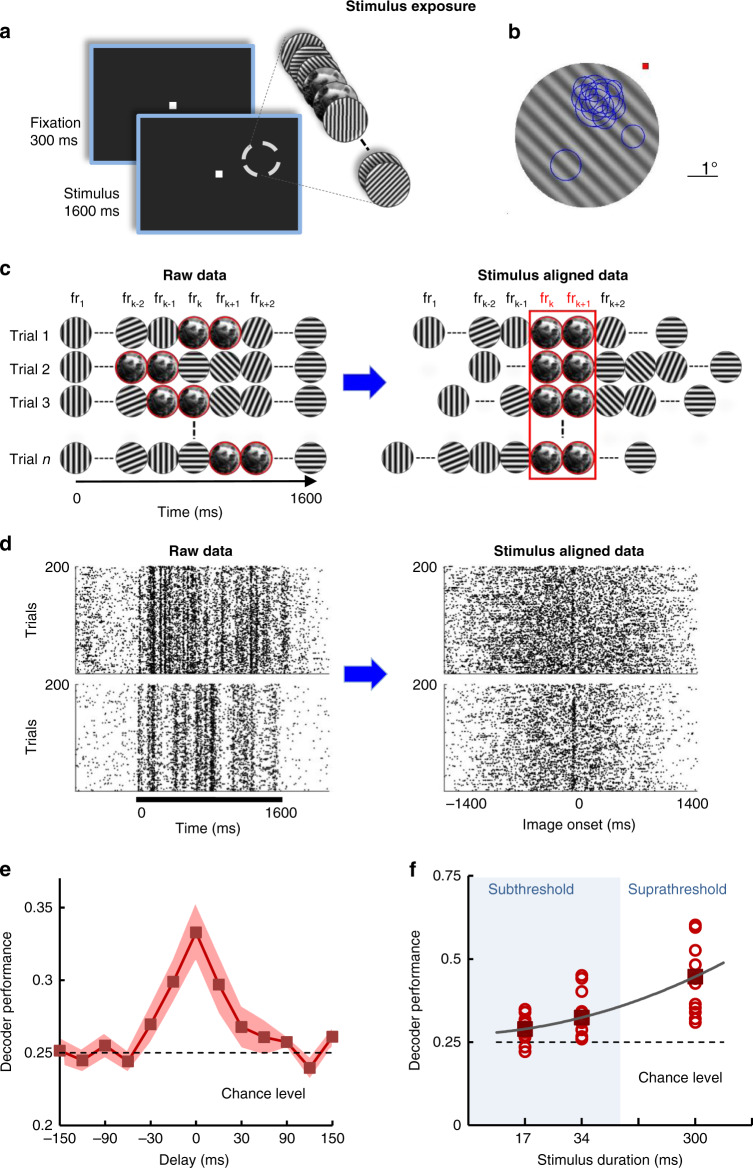
Neural populations extract stimulus information during exposure. **a** Exposure–monkeys are performing a passive fixation task while a natural image is flashed for two consecutive frames and is embedded at a random time between frames 10–86 into the movie (200 consecutive trials; the image is presented at a range of orientations, as in Fig. 3). **b** Receptive field positions (blue circles) of individual V1 neurons recorded simultaneously in a representative session shown with reference to a grating frame from the movie stimulus. **c** Cartoon describing the alignment procedure for analyzing the spike trains during exposure. Spike trains were shifted and aligned with respect to the first frame containing the image (occurring at a random time in each trial; fr_k_ represents the *k*^th^ frame in the movie). **d** Raw raster plots for example neurons recorded during stimulus exposure (left). The black bar marks the movie presentation. Same raster plots after alignment relative to the onset of the first image (right). **e** Decoder performance of a linear classifier (using firing rates calculated for a 34 ms interval) starting at different delays with respect to image onset. Neurons are reliably encoding image orientation information relative to chance level (shuffled trials) and to epochs in which the two-frame intervals contained random gratings (delays >30 ms). Error represents s.e.m, *n* = 12 sessions. **f** Decoder performance for images flashed for 17 and 34 ms, and 300 ms is significantly higher than chance level (*P* = 0.0093, *P* = 0.0048, *P* = 0.00049, respectively; Wilcoxon sign-rank test). Circles represent individual sessions (*n* = 12).

We further examined whether neural populations extract reliable information about stimuli despite the fact that they are not consciously identifiable. We thus performed daily recording sessions in which a novel image, embedded at a random time into the movie stimulus, was presented below the detection threshold (two frames; 33.33 ms), either in original form or rotated in the 5°−20° range during passive fixation (henceforth called “exposed image”, Fig. 2a; see Methods). All stimuli were displayed eccentrically (2–6°) at a location covering the receptive fields of the multiple cells recorded simultaneously (Fig. 2b; see Methods). A total of 263 single units (yielding on average 21.92 visually responsive cells per session) were isolated in two monkeys (*n* = 12 sessions; monkey C: 7 sessions; monkey W: 5 sessions). Since the timing of the image frames was randomized on a trial basis (Fig. 2c, left panel), we first rearranged the spike trains across trials with respect to the start of the first image such as to temporally align the responses elicited by the two-frame images (Fig. 2c, right panel). This changed the trial-by-trial spike matrix from movie-aligned (Fig. 2d, left panel) to image-aligned rasters (Fig. 2d, right panel).

Using a linear discriminant analysis (LDA^20^) we decoded the V1 population responses (corresponding to the four image orientations) after image alignment (see Methods). Decoder performance was computed using the firing rates during a 34 ms interval (equal to the two-frame image) starting at various times with respect to the onset of the first image frame (delay 0; performance was estimated for both negative and positive time delays; Fig. 2e). Surprisingly, decoder performance was significantly higher than chance level (0.25; *P* < 0.005, bootstrap test^21^) for intervals centered at the time of image presentation (intervals overlapping totally or partially with the image frames), and decreased rapidly to chance for time delays above 30 ms. That is, even though exposed images were perceptually unidentifiable, the population of neurons reliably distinguished them in the 34 ms interval following their occurrence in the movie sequence (classifier performance was higher when orientation difference was increased, Supplementary Fig. 2). As expected, decoder performance was elevated as the number of image frames was increased from 1 and 2 frames to 18 frames (*P* = 0.00049; Wilcoxon sign-rank test; Fig. 2f). Furthermore, the mutual information extracted by individual V1 neurons about two-frame images was significantly greater relative to trial-shuffled values (Supplementary Fig. 3a; *P* < 0.005, bootstrap test)—information sharply increased from one or two-frame (0.045 bits, *P* = 0.01 at 17 ms; 0.060 bits, *P* = 0.002 at 34 ms; Wilcoxon sign-rank test) to 18-frame stimuli (0.148 bits, *P* = 0.0005 at 300 ms; Wilcoxon sign-rank test; Supplementary Fig. 3b). Across sessions, mutual information extracted during the two-frame image exposure was correlated with decoder performance (*R* = 0.725, *P* = 0.007, Pearson correlation; Supplementary Fig. 3c).

### Subthreshold exposure increases subsequent task performance

**Fig. 3:**
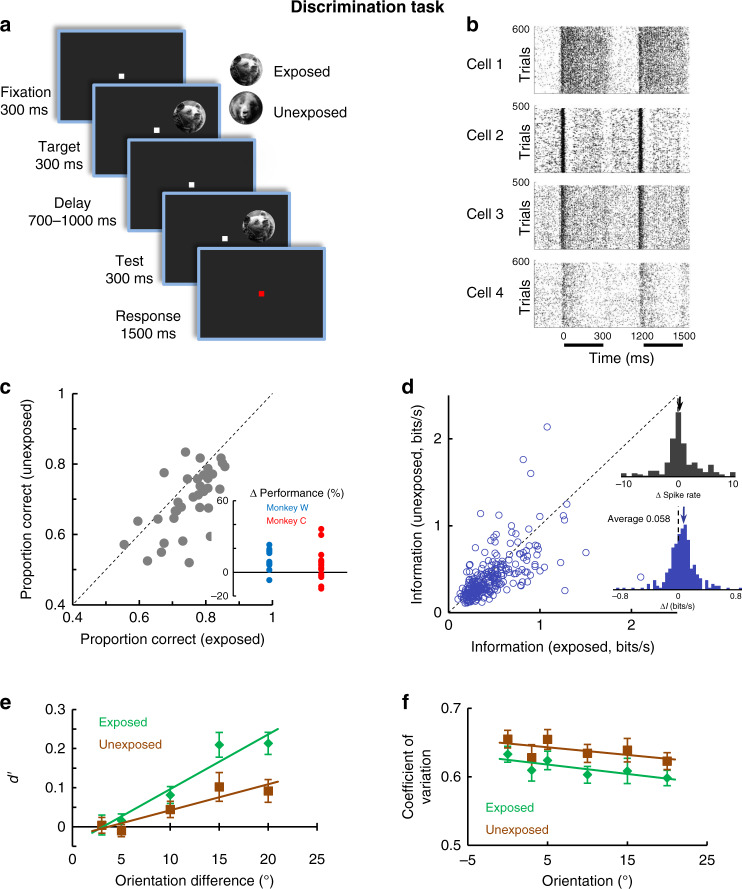
Exposure improves neural coding in V1. **a** Cartoon describing the discrimination task—monkeys are required to report whether two successively flashed images (target and test) differed or not in orientation (images were rotated by 0, 3, 5, 10, or 20°). Exposed and unexposed image trials were randomly interleaved (in 50% of trials the two images were not rotated with respect to each other). **b** Raster plots for example neurons recorded during image orientation discrimination task. The dark bars mark the stimulus presentation. **c** Proportion of total correct responses in the image discrimination task for the exposed vs. the unexposed image conditions. Each point is associated with a pair of images in one session (*P* = 0.00012, *n* = 34, Wilcoxon sign-rank test). Inset represents the change in perceptual performance in the exposed vs. unexposed conditions for each monkey. **d** Neurons transmit more information about the exposed images relative to unexposed images (*P* = 2.61 × 10^−8^, Wilcoxon sign-rank test, 300 ms, *n* = 263). Each point represents mutual information for a single neuron associated with the exposed (*x* axis) and unexposed (*y* axis) stimuli. (insets) (top) Distribution of firing rate elicited by the exposed and unexposed images. There are no significant changes in mean responses between the exposed and unexposed conditions. (bottom) Distribution of information differences, Δ*I* (*I*_exposed_−*I*_unexposed_) for the population of cells. **e** Mean neuronal sensitivity (*d’*) measured from the responses to the test stimuli (*n* = 263 neurons) is significantly greater for the exposed images (*P* = 0.0006, N-way ANOVA, *df* = 1, *F* = 11.94). **f** Mean coefficient of variation (CV) is lower for the exposed images compared with unexposed (*P* = 0.002, N-way ANOVA, *df* = 1, *F* = 9.53, *n* = 263 neurons). Error bars in all panels represent sem.

Next, we examined whether the information acquired by the population of V1 cells during subthreshold stimulus exposure is sufficient to influence perceptual performance in a subsequent behavioral task. Therefore, the exposure stage was followed by a discrimination task (Fig. 3a, b) in which monkeys signaled whether two consecutive presentations of the same image (target and test), differed or not in orientation (either identical or rotated relative to each other in the 3°–20° range). Importantly, in each session we used two images randomly interleaved across trials: one of them was the exposed image (previously presented) and the other image was novel (unexposed). The pairs of exposed and unexposed images were selected after performing image orientation discrimination experiments in humans to ensure that the two stimuli yielded similar perceptual performances (Supplementary Fig. 4). Furthermore, the orientation content, mean contrast, and luminance did not differ between exposed and unexposed images across sessions (see Methods, Supplementary Fig. 5). We used natural images rather than simple stimuli, such as oriented gratings, owing to the requirement that stimuli be novel in each session. Importantly, the same image set was used in both monkeys, i.e., exposed images for one monkey became unexposed images for the other monkey, and vice versa. Counterbalancing stimuli in each session, i.e., swapping the exposed and unexposed images within a session and repeating the exposure and discrimination experiments, or testing image discriminability before subthreshold exposure in order to compare pre and post-exposure perceptual performance, would be unfeasible. Indeed, once a stimulus is presented above threshold for hundreds of trials during image discrimination, it can no longer be used as an effective, perceptually indistinguishable stimulus capable to induce subthreshold priming.

Behavioral results (monkey C: 22 sessions; monkey W: 12 sessions) demonstrate that prior exposure to a perceptually unidentifiable image induces a robust enhancement in image discrimination performance (Fig. 3c), i.e., the proportion of total correct responses was significantly higher for exposed relative to unexposed images (0.75 vs. 0.696, *P* = 0.00012, Wilcoxon sign-rank test). Similarly, the orientation discrimination threshold was significantly lower for exposed images (6.04°) relative to unexposed (8.74°; *P* = 0.00094, Wilcoxon sign-rank test), and similar effects were found in relation to reaction times (*P* = 3.16 × 10^−11^, Wilcoxon rank-sum test; exposed: 234.37 ms; unexposed: 256.06 ms). These differences in discrimination performance were robust in each monkey (Fig. 3c inset; see Methods and Supplementary Fig. 6). Furthermore, behavioral performance associated with the two images did not vary significantly during the time course of the experiment (*P* = 0.529, one-way analysis of variance (ANOVA), *df* = 2, F = 0.65).

Additional experiments (*n* = 12 sessions) compared the strength of subthreshold stimulus exposure with suprathreshold exposure (supraliminal priming: 20 frames, 332 ms). Supraliminal exposure was followed by a discrimination task identical to that described in Fig. 3a. As expected, exposure to suprathreshold stimuli enhances the difference in behavioral performance between exposed and unexposed stimuli. Relative to subthreshold exposure, the proportion of correct responses was increased by 32.8% (exposed vs. unexposed: 0.89 vs. 0.67), whereas orientation discrimination threshold was decreased by 54% (4.74° vs. 10.3°, *P* < 0.008, Wilcoxon sign-rank test for both comparisons). This indicates that repeated supraliminal exposure increases stimulus familiarity to significantly impact behavioral performance beyond the effects of subthreshold priming.

### Subthreshold exposure increases neuronal signaling

Does exposure to perceptually unidentifiable stimuli alter their sensory representation in a subsequent supraliminal task? We first examined whether cells respond differently to the two images presented in the discrimination task, but found that while the mean firing rates to exposed and unexposed images are not significantly different (Fig. 3d, top inset and Supplementary Fig. 7, by averaging firing rates elicited by the target and test stimuli regardless of orientation, *P* = 0.0675, Wilcoxon sign-rank test), responses were changed in an orientation-specific manner such that V1 cells extracted more information about exposed images. Indeed, neurons transmitted more information in their spike counts about exposed images (0.47 bits/s) relative to unexposed (0.4 bits/s; *P* = 2.61 × 10^−8^, Wilcoxon sign-rank test, Fig. 3d), even when pooling the neurons recorded simultaneously (Supplementary Fig. 8; *P* = 0.042, Wilcoxon sign-rank test; the information difference did not vary during the time course of the session, *P* = 0.884, one-way ANOVA, *df* = 2, *F* = 0.12).

We further employed an ideal-observer analysis to measure the impact of subthreshold stimulus exposure on the sensitivity of individual cells, and hence calculated neuronal discriminability, or *d’*^22^. We found that the *d’* values for the exposed images were significantly larger than those for unexposed images (Fig. 3e, *P* = 0.0006, N-way ANOVA, *df* = 1, *F* = 11.94). In addition, we measured the neuronal precision associated with the exposed and unexposed images by calculating the coefficient of variation (CV) as the ratio of the standard deviation to mean firing rates during test presentation. We found a reduction in neuronal variability of firing rates for the exposed images, i.e., lower CVs for the images that were previously presented below threshold (Fig. 3f; *P* = 0.002, N-way ANOVA, *df* = 1, *F* = 9.53; CV did not vary significantly across orientation differences for any stimulus condition, *P* = 0.072, N-way ANOVA, *df* = 5, *F* = 2.02). Altogether, these results reveal that stimulus exposure increases the amount of sensory information transmitted by individual neurons while improving neuronal sensitivity and precision when subthreshold stimuli are subsequently presented at suprathreshold level.

### Subthreshold exposure increases sensory information during the task

**Fig. 4:**
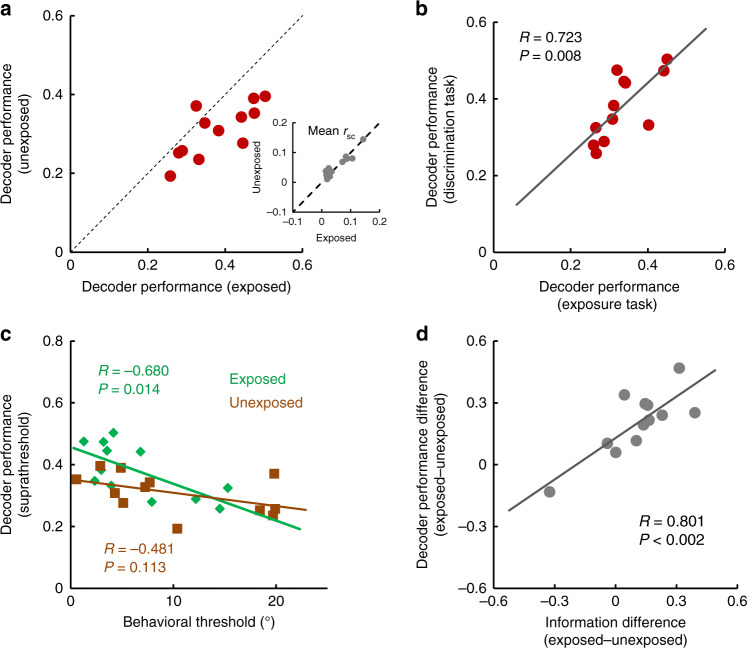
Neuronal populations extract more sensory information after subthreshold stimulus exposure. **a** The decoder performance of a linear classifier during the discrimination task is greater for the previously exposed images than for the unexposed images across recording sessions (*n* = 12, *P* = 0.0034, Wilcoxon sign-rank test). Mean firing rates from 90% of trials (sampled 50,000 times) were used as a training set; the performance of the classifier was calculated for the remaining set of trials. Inset shows the mean noise correlations (*r*_sc_) averaged across all the pairs in the recording session (*n* = 12, *P* = 0.840, Wilcoxon sign-rank test). **b**, Decoder performance during the 18-frame image presentation (300 ms images, discrimination task) is correlated with decoder performance during the two-frame image presentation (34 ms, exposure task) (*R* = 0.723, *P* = 0.008, Pearson correlation). **c** Decoder performance during the discrimination task (300-ms images) is strongly correlated with behavioral performance for exposed images (*R* = 0.680, *P* = 0.014, Pearson correlation) but not for unexposed images (*R* = 0.481, *P* = 0.113, Pearson correlation). **d** Across sessions, the increase in decoder performance (Δ*P* = (*P*_exposed_−*P*_unexposed_)/(*P*_exposed_+*P*_unexposed_)) after exposure is highly correlated with the change in mutual information (*I*_exposed_–*I*_unexposed_)/(*I*_exposed_+*I*_unexposed_) (relative change in the session-averaged mutual information of individual neurons; *R* = 0.801, *P* = 0.0017, Pearson correlation).

We further examined the link between subthreshold stimulus exposure, the information encoded in population activity, and perceptual performance. First, decoding the suprathreshold (300 ms) population response (corresponding to the five image orientations) reveals that decoder performance is elevated for exposed images compared to unexposed (Fig. 4a; *P* = 0.0034, Wilcoxon sign-rank test; both significantly higher than chance, *P* < 0.005, bootstrap test^21^). Noise correlations between neurons were indistinguishable between exposed and unexposed conditions (Fig. 4a inset and Supplementary Fig. 9a, *n* = 3200 pairs, *P* = 0.783, Wilcoxon signed-rank test) for any cortical distance between the cells in a pair (Supplementary Fig. 9b). As correlations can possibly limit the amount of available information encoded by population activity^23–28^ this indicates that the increase in decoder performance for exposed stimuli is primarily owing to changes in V1 responses across trials. Importantly, decoder performance during exposure was significantly correlated with decoder performance during the presentation of suprathreshold stimuli. (Fig. 4b; *R* = 0.723, *P* = 0.008, Pearson correlation). Moreover, for exposed images, perceptual performance in the discrimination task was significantly correlated with both decoder performance during exposure (Supplementary Fig. 10; *R* = 0.580, *P* = 0.048, Pearson correlation) and with decoder performance during the subsequent 300-ms presentation of task stimuli (Fig. 4c; *R* = 0.680, *P* = 0.014, Pearson correlation). In contrast, for unexposed images there was a significantly weaker correlation between decoder performance during the task and behavioral discrimination threshold (Fig. 4c; *R* = 0.481, *P* = 0.113, Pearson correlation). Across sessions, the increase in decoder performance (exposed vs. unexposed) was highly correlated with the increase in the amount of stimulus information extracted by individual neurons (Fig. 4d, *R* = 0.801, *P* = 0.0017, Pearson correlation).

### Increased functional connectivity after subthreshold exposure

**Fig. 5:**
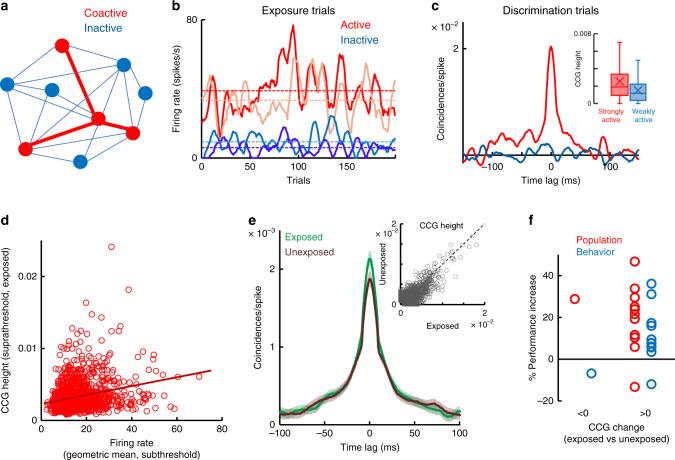
Subthreshold stimulus exposure increases functional connectivity. **a** Cartoon illustrating that exposure to two-frame stimuli activates specific neurons (active cells shown in red) leading to increased functional connectivity (red lines) when the same stimuli are presented above threshold. **b** Example trial-by-trial firing rates during the 34 ms stimulus exposure for a pair of active (red traces) and inactive neurons (blue traces). **c** Cross-correlograms (using the 300-ms stimulus interval during discrimination) for the pairs of active and inactive neurons from **b**. (inset) Average CCG peak height of strongly active and weakly active cell pairs (top and bottom thirds of all pairs, *n* = 814 each). **d** CCG peak of neurons during suprathreshold presentation of exposed images is strongly correlated with geometric mean firing rate of cells during subthreshold exposure (*R* = 0.241, *P* = 5.7  × 10^−18^, Pearson correlation). **e** Average CCG amplitude of pairs during the subsequent presentation of exposed stimuli during the task is significantly larger than CCG amplitude during the presentation of unexposed images (considering all pairs with significant CCG). The range of time lags around the central CCG bin is ±100 ms. Each cross-correlogram is shuffle corrected and normalized by mean firing rates. Error bands represent sem. (inset) Two-frame exposure increases spike time correlations between cell pairs (*P* = 3.41 × 10^−30^, *n* = 1649 pairs, Wilcoxon sign-rank test). Each dot represents the CCG peak for a pair of cells responding to exposed and unexposed images. **f** Mean changes in population decoding and behavioral performance as a function of mean changes in CCG amplitude for exposed vs unexposed stimuli. Each circle represents an individual session. The mean CCG changes have been divided based on whether they were negative or positive.

What type of mechanism could explain why stimuli presented in the absence of awareness impact sensory coding during a future behavioral task? We reasoned that these stimuli, although below the detectability and identification threshold, could nonetheless activate selected V1 neurons, and that their repeated exposure could lead to enhanced connectivity between coactive cells via Hebbian synaptic plasticity^29^. This would cause increased spike timing correlation (a measure of functional connectivity) between coactivated neurons (Fig. 5a), whereas inactive cells will not exhibit any change in spike timing correlations. This would further predict that the changes in functional connectivity induced by exposure are stimulus-dependent, i.e., they are specific to the exposed stimuli.

Across sessions, we found a diversity of neuronal responses to the two-frame stimuli ranging from inactive to active cells (Fig. 5b). During the discrimination task, when stimuli are presented above threshold, the pairs of coactive neurons exhibit significantly stronger cross-correlation compared with weakly active or inactive cells (Fig. 5c; see Methods). Indeed, the average cross-correlogram (CCG) amplitude was significantly higher for coactive cells compared with inactive cells (Fig. 5c inset; *P* = 5.34 × 10^−20^, Wilcoxon rank-sum test; comparing the top and bottom thirds of pairs after sorting them based on geometric mean firing rate). We further selected the pairs (*n* = 1248) that exhibited a significant CCG peak during suprathreshold stimulus presentation, regardless of firing rates, and found a positive correlation between the CCG amplitude during the discrimination task and neurons’ geometric mean firing rate during subthreshold exposure (Fig. 5d; *R* = 0.241, *P* = 5.7 × 10^−18^, Pearson correlation). This correlation was specific to the exposed stimuli and was significant only when firing rates were calculated from the responses to two-frame images, not to the oriented gratings during movie presentation or for baseline firing. Importantly, CCG amplitude associated with exposed stimuli was higher than that for unexposed stimuli (Fig. 5e; *P* = 3.41 × 10^−30^, *n* = 1649 pairs, Wilcoxon sign-rank test; comparing all the pairs for which the CCGs had a statistically significant peak for exposed and unexposed images, regardless of firing rates).

Although we did not find a session by session direct relationship between the increase in performance and CCG peak height, most of sessions are associated with increased decoder and behavior performance for exposed vs. unexposed images, as well as a CCG increase in the same stimulus conditions. Accordingly, population decoding and behavioral performance during the discrimination task were elevated in 88.3% of sessions in which we found an increase in mean CCG amplitude for exposed stimuli relative to unexposed (Fig. 5f). Furthermore, CCG amplitude was correlated with the geometric mean of mutual information of the coactive pairs of neurons (Supplementary Fig. 11). Taken together, these analyses provide support for our hypothesis that exposure to subthreshold stimuli increases the connectivity between the subset of neurons activated by the exposed stimuli. Further, these cells exhibit elevated spike timing correlations during the task when the same stimuli are presented above the detectability threshold to contribute to enhanced discrimination performance.

### Control experiments

One concern is that our results might have been influenced by an inherent bias, or preference toward exposed stimuli. For instance, V1 neurons could have extracted more information from exposed stimuli simply because these stimuli were more informative. Although this is unlikely to be the case since exposed and unexposed stimuli were swapped across animals and owing to our multiple stimulus controls (Supplementary Figs. 4–5), we nonetheless performed additional recordings to directly address this issue. That is, 45 weeks after the end of our original experiments (Figs. 2–5), we selected the image pairs associated with the largest exposure-induced effects on mutual information and decoder performance. We reasoned that 45 weeks would be sufficient to erase any image familiarity since initial stimulus exposure^16^. Nonetheless, contrary to our main results, there was no evidence that V1 neurons extract more information from the originally exposed stimuli (*n* = 90 cells, four sessions). That is, neurons had identical neuronal sensitivity, *d’* (Supplementary Fig. 12a; *P* = 0.445, N-way ANOVA, *df* = 1, *F* = 0.58), coefficient of variation, *CV* (Supplementary Fig. 12b; *P* = 0.236, N-way ANOVA, *df* = 1, *F* = 1.4), spike time synchrony (Supplementary Fig. 12c, *P* = 0.820, Wilcoxon sign-rank test), and amount of extracted information associated with the two groups of images (Supplementary Fig. 12d, *P* = 0.969, Wilcoxon sign-rank test). Furthermore, decoding population activity reveals that, in the absence of exposure, classifier performance is not different between the two sets of images (Supplementary Fig. 12e; *P* = 0.562, Wilcoxon sign-rank test). The changes in behavioral and neuronal performance associated with exposed and unexposed stimuli were unrelated to oculomotor variables, such as changes in eye movements or pupil size (see Methods and Supplementary Fig. 13; *P* = 0.098, Wilcoxon sign-rank test). Altogether, these results indicate that the effects of exposure on stimulus encoding are not owing to stimulus-specific inherent differences in the amount of information extracted by V1 neurons.

## Discussion

We demonstrated that natural images briefly presented below the detectability threshold are encoded by V1 cell populations such as to improve perceptual and neuronal performance when the same stimuli are subsequently presented above threshold in the context of a behavioral task. Indeed, exposure to subthreshold, behaviorally irrelevant, stimuli in the absence of awareness improves stimulus discriminability and perceptual performance while increasing the amount of sensory information extracted by V1 population activity, and the sensitivity and precision of individual cells. Thus, subthreshold stimuli activate neuronal networks involved in perception, and this activation contributes to subsequent changes in sensory representation. Prior studies of priming were performed in humans^1–5,7–11^, but whether subthreshold stimuli change the sensory representation at single cell or population level to control behavior has been unknown. Our results demonstrate that visual cortex is endowed with the remarkable capacity of extracting environmental information even though this information is presented below the level of awareness and is behaviorally irrelevant.

Our analysis of neural recordings indicates that a Hebbian-type mechanism could underlie the improvement in perceptual processing following exposure. Indeed, exposure to subthreshold stimuli elicits weak responses in V1 that can nonetheless be decoded from population activity. Decoder performance was slightly higher than chance, although significantly lower than that associated with suprathreshold stimuli, which explains why the image information contained in subthreshold stimuli is perceptually indistinguishable. Further, subthreshold stimuli preferentially activate a subset of neurons that form a cortical ensemble^29^ in a stimulus-specific manner. Cells within this ensemble exhibit an increase in functional connectivity associated with exposed stimuli (Fig. 5e). That is, subthreshold exposure induces a “familiarity” effect for exposed stimuli, which are subsequently encoded more accurately than novel, unfamiliar stimuli.

Attention could be a possible candidate to explain the differences in perceptual and neuronal performance induced by repeated exposure to subthreshold stimuli. Indeed, more attention elicited by exposed images is expected to cause an increase in perceptual and neuronal performance. However, this is unlikely to be the case. First, subthreshold stimuli could not be identified as the image content was perceptually imperceptible. Therefore, both the “exposed” and “unexposed” stimuli during image discrimination were novel to the animal. Furthermore, our image analysis has ensured that all the images in our set were equalized for mean luminance and orientation content (Supplementary Fig. 5). Importantly, when we swapped the exposed and unexposed stimuli across animals, we found robust priming effects in each animal (Fig. 3c, inset and Supplementary Fig. 6). Altogether, these data rule out that exposed images elicit more attention. Second, the changes in neuronal responses after subthreshold exposure are inconsistent with attention as a major variable explaining our results. Indeed, we did not find statistically significant changes in mean responses and noise correlations, two of the major effects of attention on visual cortical responses^30,31^, when exposed images were presented. Third, our control experiments performed 45 weeks after the original priming experiments demonstrate a lack of difference in neuronal responses associated with previously exposed and unexposed stimuli (Supplementary Fig. 12), hence arguing against attention as a confounding variable.

One could reason that the choice of our task—image discrimination—might not have been ideally suited for electrophysiological recordings in V1. However, the use of complex stimuli, such as natural images, rather than simple stimuli, such as orientation, presents several advantages for priming studies: (i) one important requirement in our experiments is that stimuli should be novel in each session, which would be impossible to satisfy using simple stimuli that are both limited in range and prone to elicit generalization effects in V1 when presented in a task context. Investigating priming using stimuli the animal has experienced in recent past would render the comparison between the effects associated with exposed and unexposed stimuli difficult to interpret. (ii) Natural scenes have been demonstrated to be well suited for visual psychophysics and, in addition, they elicit vigorous responses in individual neurons and populations^32,33^. (iii) The fact that natural images exhibit local spatial correlations^34^ ensures monotonic changes in neuronal firing rates with increasing image rotation angle (as in Fig. 3e), which subsequently allows us to relate neuronal and behavioral performance.

What could be the functional benefit of encoding stimuli presented below the level of awareness? Over the past several decades, attention studies have demonstrated that the brain filters out weak, irrelevant information by controlling local excitatory and inhibitory mechanisms via top–down projections. These mechanisms play a crucial role by allowing the brain to select stimuli that are functionally relevant. Although priming induced by subthreshold stimuli may seem to be a detrimental mechanism, especially in the face of the constant daily bombardment with unwanted information, this type of unsupervised adaptation may be the remnant of a primitive pre-attention system using the mere frequency of occurrence to change stimulus representations even when sensory inputs are weak^2,3^. Future studies will elucidate how the effects of subthreshold stimulus priming observed in early sensory areas are transmitted and processed in higher brain regions to influence decision making.

## Methods

### Ethics statement

Two male rhesus monkeys (*Macaca mulatta*) were used in the experiments. All experiments were performed in accordance with protocols approved by the U.S. National Institutes of Health Guidelines for the Care and Use of Animals for Experimental Procedures and were approved by the Institutional Animal Care and Use Committee at the University of Texas Health Science Center at Houston. Human research participants (15 adult subjects aged 21–46 years old) had normal or corrected to normal vision and were only used in preliminary or control behavioral experiments. This study was approved by the local institutional ethics committee.

### Behavioral paradigm

Stimuli were presented binocularly on a computer screen of black background at a viewing distance of 90 cm. In all the tasks performed in this study, subjects (monkeys or humans) were required to fixate on a centrally located fixation point (0.4 deg size) within a 1-deg fixation window while stimuli with a diameter of 5–8 deg were presented at 4–8 deg eccentricity. The location and size of stimuli was the same within each session and covered the multiple receptive fields of all the recorded cells (Fig. 2b). Receptive fields for each cell were mapped at the beginning of each recording session using reverse correlation stimuli. Each trial required that monkeys maintain fixation while the fixation point was present on the screen. The trial was aborted if monkeys broke fixation or fixation instability exceeded 0.25 deg. To ensure stable fixation, eye position was continuously monitored using an eye tracker system (EyeLink II, SR Research Ltd.) operating at 1KHz. Monkeys were required to hold a metal lever at the beginning of the trial and maintain contact for the duration of the trial unless a behavioral response was needed. Correct behavioral responses were rewarded with three drops of juice.

### Detectability threshold

In preliminary image detectability tests (Fig. 1a) a movie stimulus consisting of a sequence of 96 circular sinusoidal gratings (eight equidistant orientations randomly flashed at 60 Hz) was presented for a total duration of 1600 ms. In 50% of the trials, a variable number of frames (between 2 and 15 consecutive frames presented at a random time between frames 18 and 79) were replaced by a circular grayscale natural image of identical size and mean luminance as the gratings. Monkeys were required to signal whether the image was present in a trial by releasing the lever immediately at the end of the movie stimulus presentation. By varying the number of image frames on a trial-by-trial basis, behavioral results were used to determine the image detectability threshold (Fig. 1b). Psychometric curves and thresholds were obtained by averaging the performances associated with each number of consecutive image frames in all sessions (*n* = 16, 6) and fitting the psychometric curve to a Weibull function^35,36^. The discriminability threshold was calculated by setting the threshold at *d’* = 1, while taking into account the false alarm rates^35^.

### Image identification threshold

We conducted a forced-choice saccade task in which monkeys reported image identity. This allowed us to test whether animals are able to perceive the image content and consciously identify the subthreshold (two frames) or suprathreshold (more than five frames) stimuli. After a 300 ms fixation period, monkeys were presented the same 96 frames movie stimulus used in the detection task, except that in each trial one of two images was embedded within the movie (between 2 and 40 consecutive image frames; trials were randomly interleaved). A control stimulus with 0 image frames (0 ms) had the same probability of occurrence as any of the other frame lengths. At the end of stimulus presentation, the two images appeared on the screen (Fig. 1c). Monkey were required to maintain fixation (150 ms) until the fixation point turned red, and then performed a saccade towards the image they had previously seen. Monkeys were allowed to make a saccade within 700 ms, and once they made the saccade, they maintained fixation for at least 700 ms. Correct responses were rewarded with juice while failure to make a saccade aborted the trial. Proportion of correct responses were averaged for each number of frames and the results were fitted with an exponential function (Fig. 1d). The discriminability threshold was calculated by setting the threshold at *d’* = 1^35^.

### Additional controls in humans

We performed additional control psychophysical experiments in humans that confirmed that the image content (category) was not identified when two-frame images, identical to those used in the monkey experiments, were used in a forced-choice category identification experiment. Subjects (*n* = 9) were required to classify the image (Supplementary Fig. 1a) in different classes, e.g., bird or mammal, 50 trials each. In this experiment, subjects failed to respond above the chance level (proportion correct responses = 0.49, *P* = 0.5781, Wilcoxon sign-rank test; Supplementary Fig. 1b). This result also confirmed that even if a small discontinuity might have been occasionally noticed within the movie stimulus, the content of that information was never consciously processed. A second control experiment (*n* = 15 subjects) required the identification of the two-frame stimulus image (embedded within a 1600-ms movie), identical to the stimulus in Fig. 2a and Supplementary Fig. 1a, from a set of five images displayed on the screen for 5 s immediately following the movie offset (multiple sets of five images were used, randomly repeated three times each, with the test image randomly selected from the set); subjects, again, failed to respond above chance (proportion correct responses = 0.21, *P* = 0.8374, Wilcoxon sign-rank test).

### Subthreshold stimulus exposure

Repeated two-frame presentations of a natural image—the exposure task—was conducted before each image orientation discrimination session. Monkeys were required to fixate on a 0.4 deg central fixation spot while a movie stimulus consisting of 96 full contrast circular sinusoidal oriented gratings spanning 0–180° (eight gratings, 22.5° orientation step, 12 repetitions) was presented at the receptive field location. The gratings were displayed at a frequency of 60 Hz for a total movie duration of 1600 ms. Within the movie, two consecutive frames (33.33 ms), randomly inserted between frames 10 and 86, were replaced by a grayscale natural image (Fig. 2a). Masked high-contrast images were used because they elicit strong responses from V1 neurons, necessary to ensure reliable measurements, such as mutual information, decoder performance, and CCGs and because the same stimuli were subsequently presented in discrimination experiments. The exposed image was presented in a randomly interleaved manner across trials either at its original orientation or rotated in the 5–20° range, i.e., the same image rotations used in the subsequent discrimination task. Each exposure session consisted of 200 trials. In each session we used a different, novel exposed image. No behavioral response was required in this task.

### Image orientation discrimination task

Several minutes after the completion of the image exposure phase, animals were required to complete an image orientation discrimination task using two images in each session: one of the images was the exposed image, whereas the other image was a novel image that monkeys have never seen before (Fig. 3a). Images were adjusted in mean luminance so that they matched each other, as well as the mean luminance of the grating stimuli. The size and location of the stimuli were identical to those used in the exposure phase. In each trial, one of the images was flashed twice (second image was either identical to the first or rotated counterclockwise; the number of match and non-match trials was identical) for 300 ms, separated by 700–1000 ms random delay interval. Each session had between 480 and 640 trials. Since the two monkeys had slightly different overall performance levels, image rotations were 3°, 5° 10°,and 20° for monkey C, and 5°, 10°, 15°, and 20° for monkey W. Monkeys were required to signal whether the two images were identical or different (rotation) by releasing/holding the lever. Match and non-match trials were randomly interleaved.

### Analysis of behavioral data

Discriminability index (*d’*) for each exposed and unexposed image (Supplementary Fig. 6a—monkey W; Supplementary Fig. 6b—monkey C) were computed as the difference between the z-transforms of the hit (*H*) and false alarm (*F*) rates: *d’* = *z(H) − z(F)*^33^ for each orientation difference and averaged over all sessions. Behavioral performance for the two monkeys was measured by calculating the proportion of correct responses for all orientations (Supplementary Fig. 6c—monkey W, *P* = 0.0024; Supplementary Fig. 6d—monkey C, *P* = 0.0090; Wilcoxon sign-rank test), the orientation discrimination threshold (Supplementary Fig. 6e—monkey W, *P* = 0.0122; Supplementary Fig. 6f—monkey C, *P* = 0.024; Wilcoxon sign-rank test), and the response reaction time. For each of the two monkeys all these measures indicated the same result—an increase in behavioral performance after subthreshold image exposure. The discrimination threshold was calculated by fitting the psychometric curves to a Weibull function^36^ and setting the threshold at *d’* = 1, while taking into account the false alarm rates^35^. Response reaction times were calculated as the time interval between the end of the test image presentation and the release of the response lever.

As a control, the same image orientation discrimination task was performed by human subjects (Supplementary Fig. 4) in the absence of exposure (*n* = 11). None of the parameters described above were changed except for image rotation angle (5–30° range, owing to the unfamiliarity with the task and the grouping of three image pairs in one session). There was no significant difference in performance between the “exposed” and “unexposed” images (*P* = 0.687, Wilcoxon sign-rank test).

### Control experiment after exposure

Additional controls were performed 45 weeks after the main experiment. The purpose of this control was to ensure that the differences in the amount of information that individual neurons and populations transmit about exposed and unexposed stimuli is not owing to an inherent bias, or preference that animals might have exhibited for the exposed stimuli, or simply because these stimuli are more informative. The control consisted of a passive fixation task in which selected grayscale natural images (5–8 deg diameter), previously used in the main exposure/discrimination experiments were displayed eccentrically (4–8 deg) at same locations where exposed and unexposed images were originally presented. Previously exposed and unexposed images were randomly interleaved and flashed for the same duration (300 ms) and range of orientations (between 0 and 20°), exactly as in the original priming experiments) at least 200 times in a given session. We recorded a total of 90 neurons (*n* = 4 sessions, 3–6 image pairs per session, 21 total image pairs, *n* = 486 data points) at the same cortical location as that where the previously recorded population of neurons were shown to exhibit pronounced effects associated with the two-frame image exposure (Figs. 2–5). All these neurons had receptive fields covering the spatial location where the exposed and unexposed images were presented in the original experiments.

### Image selection

Images were grayscale (256 grey levels) and were extracted from a high-resolution, commercial photo-CD library. Both images used in a given session were carefully selected to have equivalent attributes, such as mean luminance, contrast, and orientation distribution. We computed the orientation content of each image by applying a Sobel filter to each original image (Supplementary Fig. 5a^34,37^), which generates an orientation filtered image (Supplementary Fig. 5b) containing orientation information at each pixel location. We then calculated the orientation magnitude histogram for the exposed and unexposed images (Supplementary Fig. 5c). We ensured that the image pairs selected in a session have similar orientation distributions (number of peaks above the mean in the orientation distribution of each image) and orientation selectivity index^32,37^. This index was calculated as the sum of orientation bins above the mean in the orientation magnitude histogram divided by the number of bins above the mean (Supplementary Fig. 5d). In addition, we repeated the same measure considering only the bins within ±5° of the cardinal orientations (Supplementary Fig. 5e). For both of these measures there was no significant difference between the two sets of images (*P* = 0.587, *P* = 0.647, Wilcoxon sign-rank test). Importantly, the same pair of images was used in both monkeys, but the exposed and unexposed images were swapped; for instance, the “exposed” image for Monkey W became the “unexposed” image for monkey C, and vice versa.

### Electrophysiological recordings

Extracellular recordings in primary visual cortex (area V1) were performed during both the exposure phase (Fig. 2d, left panel) and the subsequent discrimination task (Fig. 3b) for the same population of neurons held stable throughout each daily session. We used a combination of laminar multi-contact electrodes and microelectrodes mounted on a 1 mm spaced custom grid (Crist Instruments) lowered inside a 19-cm recording chamber. Recording sites were selected on the basis of the quality of the signal (signal-to-noise ratio) and responsiveness to visual stimuli. Electrodes were advanced transdurally through stainless steel guide tubes using a computer controlled microdrive (NAN Instruments). Single-contact electrodes were tungsten microelectrodes with impedances 1–2 MΩ at 1 kHz (FHC Inc.). The laminar electrodes (U-probe, Plexon Inc) consisted of a linear array of 16 equally spaced contacts (100 µm inter-contact spacing). Each electrode contact was 25 µm in diameter and platinum iridium coated. The impedance at each contact was 0.3–0.5 MΩ. In separate recording sessions, we used either one or two laminar electrodes along with several single-contact electrodes. Real-time neuronal signals recorded from both electrode types (simultaneous 40 kHz A/D conversion on each channel) were analyzed using the Multichannel Acquisition Processor system (MAP system, RASPUTIN v2 HLK2 software, Plexon Inc). Single-unit recordings were amplified, filtered, viewed on an oscilloscope and heard through a speaker.

### Data analysis

Individual neurons were isolated by sorting spike waveforms using Plexon’s offline sorter program. A total of 263 single-unit cells were isolated in the two monkeys (visually unresponsive or low responsive cells, i.e., average firing rate < 1 spk/s, as well as unstable neurons that exhibited more than 20% change in peak firing rates were removed from the analysis) across 12 recording sessions (monkey C: 7 sessions; monkey W: 5 sessions). We recorded on average 21.92 visually responsive cells per session with no significant difference between the two monkeys (*P* = 0.2879, Wilcoxon rank-sum test; monkey C: 23 cells/session; monkey W: 20.4 cells/session). For each session, the same neurons were used in all the analyses associated with the exposed (both exposure and discrimination task) and unexposed images.

Extracting neurons’ responses to the masked 2-frame exposure images was not straightforward owing to the short image duration and random position in the movie as well as strong responses to orientation gratings embedded within the movie (Fig. 2c, d, left panels). We first calculated the response latency of each neuron and aligned the spike trains of the multiple neurons recorded simultaneously. Next, we aligned the spike trains for each trial by the onset of the first image frame, hence shifting the spike trains by a number of frames equal to the index of the first image frame (Fig. 2c). Thus, the resulting spike time matrix changed from grating-aligned (Fig. 2d, left panel) to image-aligned (Fig. 2d, right panel), in order to allow us to extract the neural responses triggered by the two-frame images. After alignment, the responses before and after image presentation were used to quantify the baseline level, used in calculating mutual information (or determining the chance level for decoder performance). Figure 2e and Supplementary Fig. 3a not only revealed that the V1 population encoded the images flashed for 33.3 ms, but also confirmed the accuracy of the alignment procedure described above.

For above threshold images, mutual information, *d’*, coefficient of variation, CCG, and decoder performance were calculated for the 300-ms presentation of the test image during orientation discrimination (starting 35 ms after stimulus onset). The target image always had a fixed orientation. Neural discriminability^22^, or *d’*, was calculated as the difference between the firing rate for each image rotation *R*_i_ and the firing rates for original unrotated image *R*_*0*_, normalized by their variances *SD*_*i*_ and *SD*_*0*_

1 $$d'_i = \frac{{R_0 - R_i}}{{\surd \left[ {\left( {SD_0^2 - SD_i^2} \right)/2} \right]}}$$

*d’* was computed for each test stimulus, and all orientation differences were included in the analysis.

To quantify how much information neural responses carry about image orientation, we calculated the mutual information between the set of orientations and the set of associated firing rates elicited by the test stimuli. For discrete variables, mutual information *I* of two variables *x* and *y* is defined based on their joint probabilistic distribution *p(x,y)* and the respective marginal probabilities *p(x)* and *p(y)*:

2 $$I\left( {x,\;y} \right) = \mathop {\sum}\nolimits_{i,\;j} {p( {x_i,\;y_j} ){\mathrm{log}}\frac{{p( {x_i,\;y_j} )}}{{p( {x_i} )p( {y_j} )}}}$$

The calculation of mutual information was done by using the ‘mutual information computation’ MATLAB package^38^ available at: http://www.mathworks.com/. Information was calculated for the 1 and 2 frames (17 ms and 34 ms intervals during exposure task) and 18 frames (300 ms–discrimination task) images. Two values of the mutual information were computed for each neuron during the discrimination task: Information “exposed” and Information “unexposed” corresponding to the exposed and unexposed images. Our results were statistically significant for both monkeys (as described in the text) and invariable to various data manipulations, such as: pooling the image orientations into small vs. large orientation data sets, dividing into early vs. late trial blocks, and separating neurons depending on different recording depths on the laminar electrode.

We used a multiclass LDA algorithm implemented in Matlab to determine the effect of exposure on neuronal population activity. This supervised learning method uses a subset of trials as training set and determines the class of the other subset of trials by maximizing the ratio of between-class variance to within-class variance in any particular data set thereby guaranteeing maximal separability. The decoder was trained using the mean firing rates from 90% of the trials (sampled 50,000 times), and its performance to distinguish the different image orientations was tested for the remaining 10% of trials (by comparing its prediction in each trial against the real orientation of the image displayed). The results were confirmed by using Naive Bayes and k-nearest neighbor approaches. The significance of the decoding performance was assessed using methods previously described^21,39^, i.e., calculating the *p* value by adding up the probabilities of getting the same or more hits by chance. Decoder performance was calculated both for the 1 and 2 frames (17 ms and 34 ms intervals–exposure task) and 18 frames (300 ms–discrimination task) images. We found that classifier performance was statistically significant in 10 out of 12 recording sessions. We then compared decoder performance between the subthreshold and suprathreshold presentation of the same images (four out of five images orientations were used for suprathreshold images in this comparison), and between both the exposed and unexposed suprathreshold data sets. Noise correlations^23,25^ were calculated for pairs of simultaneously recorded neurons in each session, separately for the 300-ms exposed and unexposed test images.

CCGs were computed by sliding the spike trains of each cell pair and counting coincident spikes within 1 ms time bins for each stimulus condition and pair of neurons, normalized by the geometric mean spike rate, and corrected for stimulus-induced correlations by subtracting an all-way shuffle predictor^39,40^. CCGs were smoothed with a 2 ms Gaussian kernel and fitted to a Gaussian function. The significance of the CCG peaks was assessed for peaks (−50 to 50 ms interval) that exceeded three standard deviations of the noise (tail) level at time lag from −400 to −250 ms and from 250–400 ms. Mean CCGs were obtained by averaging CCGs for all the pairs for each of the exposed and unexposed stimuli (separately).

Eye position throughout the trial was convolved with a low-pass linear finite impulse response filter with a 50 Hz cutoff frequency. Microsaccades were identified as deflections of eye position for which eye velocity exceeded 10 deg/s for at least 10 consecutive ms, and eye acceleration exceeded 1000 deg/s^2^ during a 40 ms interval centered at the maximum of the eye velocity. Successive microsaccades separated by <30 ms were considered as a single eye movement. Pupil area (Supplementary Fig. 13) was recorded by the eye tracker system (EyeLink II, SR Research Ltd.), and was represented in arbitrary units separately for the exposed and unexposed image trials.

### Statistical analysis

Statistical significance was assessed using two-tailed nonparametric tests. Specifically, for comparing the exposed and unexposed conditions, Wilcoxon signed-rank test was used. We compared either the values for the whole neuronal populations recorded across sessions (e.g., mutual information of each neuron) or the variables computed for each session (e.g., behavioral, decoder performance). In case of unmatched observations with unequal sample size (e.g., reaction times involving a different number of trials), Wilcoxon rank-sum test was used. We chose these tests rather than parametric tests, such as the *t* test, for their greater statistical power (lower type I and type II errors) when data are not normally distributed. For populations involving two or more independent variables, a N-way ANOVA test was used. Bootstrap tests comparing the results with shuffled data (decoder performance significance) used 50,000 iterations.

### Reporting summary

Further information on research design is available in the Nature Research Reporting Summary linked to this article.

## Supplementary information

Supplementary Information

Reporting Summary

## Data Availability

The data upon which this study was based are available from the corresponding author upon reasonable request.

## Code availability

Data analysis was performed using MATLAB (Mathworks). The code upon which this study was based are available from the corresponding author upon reasonable request.
