# Supplementary material for: Perceptually unidentifiable stimuli influence cortical processing and behavioral performance
Source: Nat Commun. 2020 Nov 30;11:6109. doi: 10.1038/s41467-020-19848-w (PMC7705662; doi:10.1038/s41467-020-19848-w)
Supplement: Supplementary file 1 — Supplementary Information [file 41467_2020_19848_MOESM1_ESM.pdf]

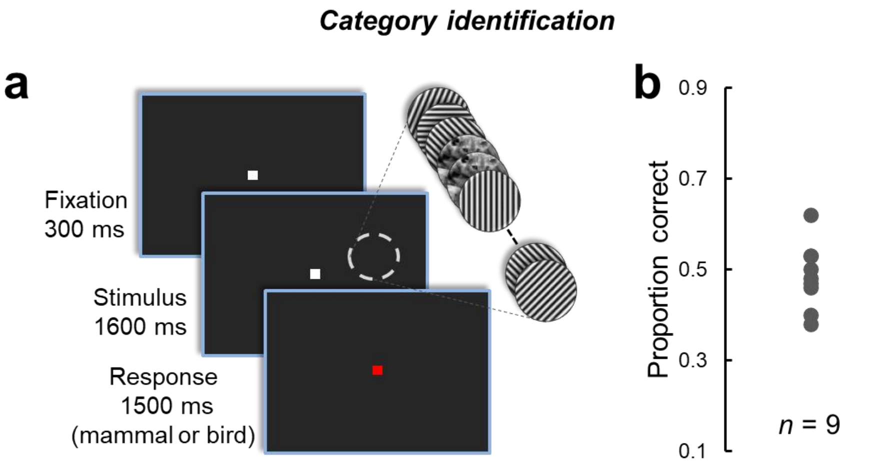

Supplementary Figure 1. Control experiment involving subthreshold stimuli – category identification. a, Experimental setup. Human subjects (n = 9) were asked to categorize the type of image (mammal or bird) that was displayed for 2 consecutive frames during the presentation of the movie stimulus (identical characteristics as the stimulus used in our main 'exposure' experiment). We chose 2 frames in order to directly relate the results to the experiments in **Fig. 2a**. **b,** Behavioral results – dots represent the proportion correct identifications across subjects. The proportion correct responses was not significantly different from chance level (0.49, P = 0.5781, Wilcoxon sign-rank test).

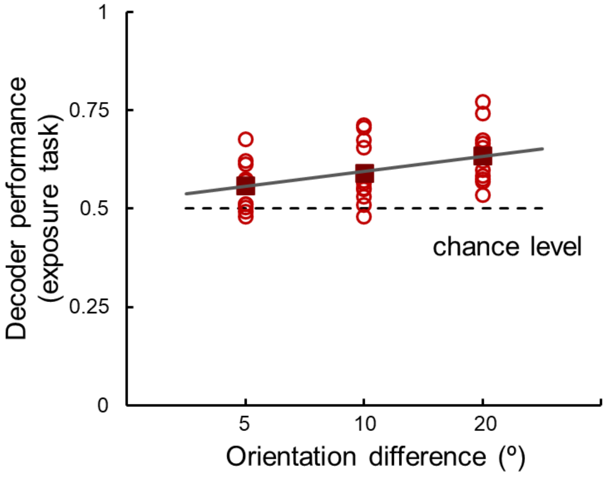

Supplementary Figure 2. Decoder performance during exposure task. Decoder performance for pairs of 2 image orientations (out of 4 images/conditions) for the 34 ms image exposure. Across sessions, decoder performance was significantly different from chance (*P* = 0.01, 0.0015, *P* = 0.0005, Wilcoxon sign-rank test) for any orientation difference between the images (0.558 at 5º, 0.590 at 10º and 0.635 at 20º; chance level for the 2-condition decoder is 0.5), and increases with orientation difference (*P* = 0.012, Wilcoxon sign-rank test, between the 5º and 20º difference).

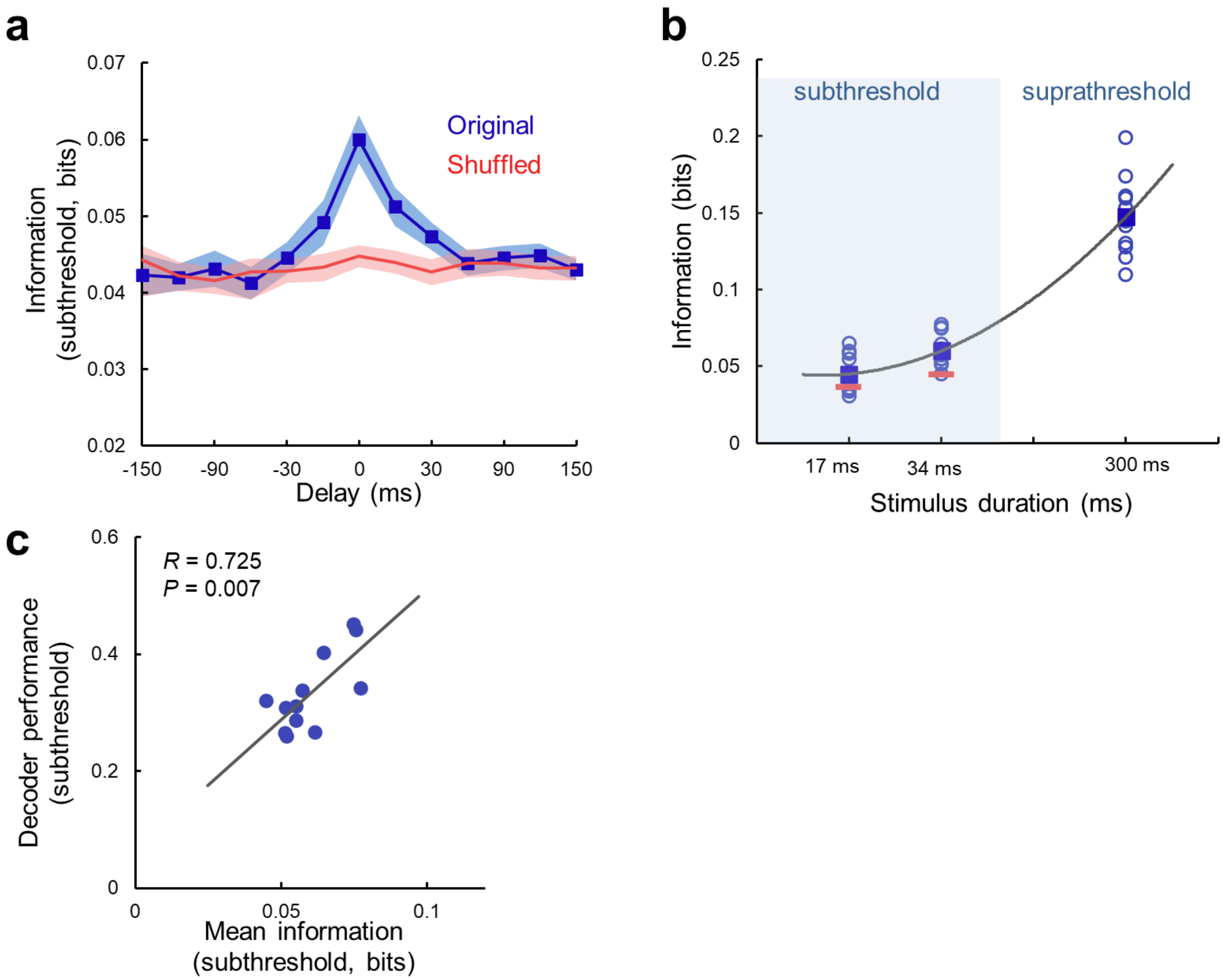

Supplementary Figure 3. Mutual information during exposure task. a, Mutual information (using the firing rates during a 34 ms interval) starting at the time of the first image frame (delay 0) and at various other times in the aligned stimulus (see **Fig. 2c,d**, right panels). Individual V1 neurons are able to encode image orientation information compared with chance (shuffled trials) and random orientation gratings (delays greater than 30 ms; see fr_{k-1}, fr_{k+2}, etc. in **Fig. 2c**, right panel and **Fig. 2d**, right panel; n = 12 sessions). **b,** Mutual information rapidly increases from 1 and 2-frame images (17 ms - 0.045 bits, P = 0.01; 34 ms - 0.060 bits, P = 0.002, Wilcoxon sign-rank test, n = 12 sessions, exposure task) to suprathreshold image presentation (300 ms, discrimination task - 0.148 bits, P = 0.0005, Wilcoxon sign-rank test, n = 12 sessions). Mutual information during suprathreshold stimulus presentation is significantly higher than that extracted from subthreshold stimuli. Red lines show the chance (shuffled) values. **c,** Across sessions (n = 12), mutual information during exposure (34 ms interval) is correlated with decoder performance calculated for the same time interval (R = 0.725, P = 0.007, Pearson correlation). Error bars or bands in all panels represent sem.

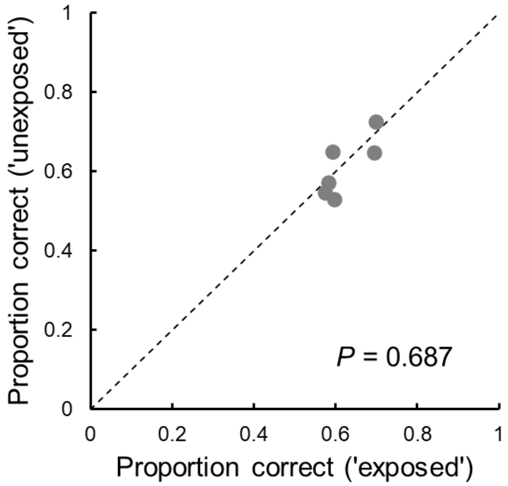

Supplementary Figure 4. Discrimination task in absence of exposure. Behavioral results of an image discrimination task identical to that in **Fig. 3a**, but in the absence of exposure. This control experiment was performed by human subjects (n = 11) using 6 of the image pairs from the main experiment. There was no significant difference in performance between ‘exposed’ and ‘unexposed’ images (P = 0.687, Wilcoxon sign-rank test).

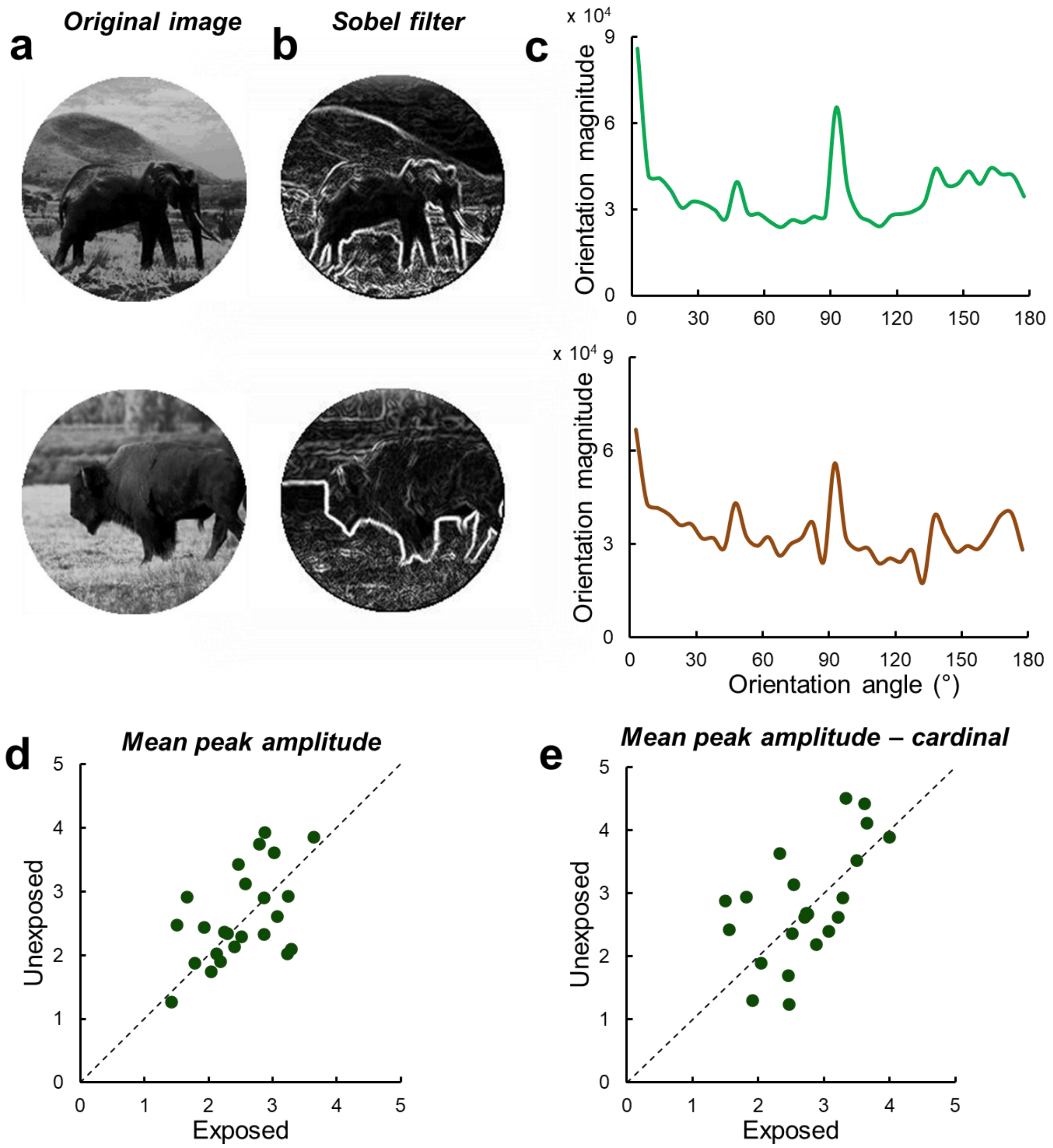

Supplementary Figure 5. Image analysis. a, Examples of one set of images used in the exposure/discrimination experiment. **b,** Orientation filtered images obtained after applying a Sobel filter to the original scenes. **c,** Orientation magnitude histogram computed from images in panel **b**. **d,** Mean peak amplitude for the exposed and unexposed images, calculated as the sum of orientation bins above the mean (taken from the orientation magnitude histogram) divided by the number of bins above the mean. Our analysis demonstrates that across images there is no difference in the strength of orientation signals between the exposed and unexposed stimuli (P = 0.59, Wilcoxon sign-rank test, n = 24). **e,** Mean peak amplitude for the bins within ±5° of the cardinal directions (the sum of orientation bins within ±5° of the cardinal directions above the mean in the orientation magnitude histogram divided by the number of bins above the mean; P = 0.65, Wilcoxon sign-rank test, n = 24).

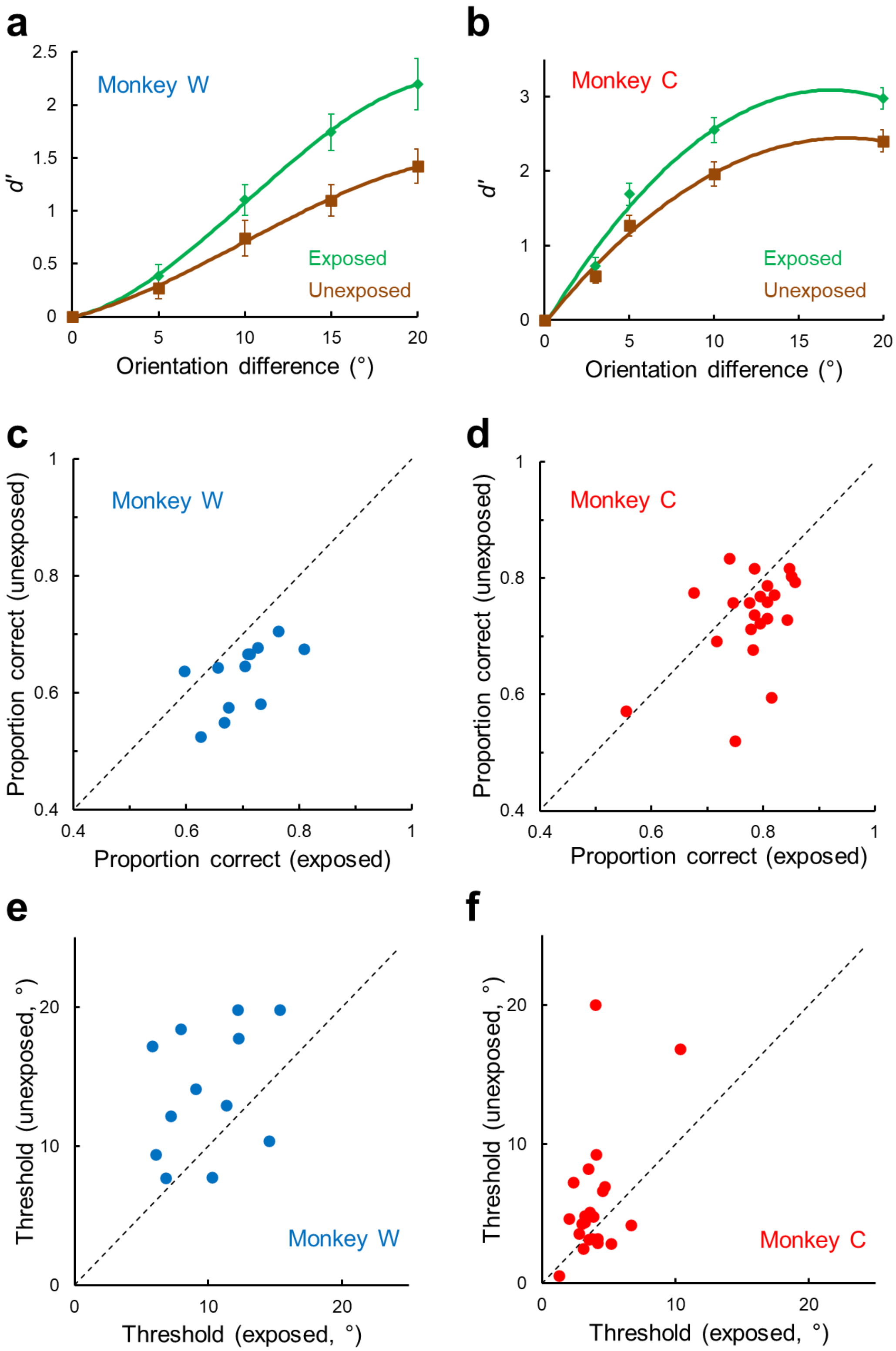

Supplementary Figure 6. Behavioral performance for each monkey. a-b, Behavioral discrimination performance (*d'*) curves for the exposed and unexposed images in each monkey. Error bars represent sem. **c-d,** Proportion of total correct responses in the image orientation discrimination task for the exposed vs. unexposed images. Each point represents a pair of images used in one session (n = 12 for monkey W, and n = 22 for monkey C). **e-f,** Discrimination threshold (calculated at d' = 1) for the same pairs shown in panels **c-d** during image orientation discrimination task for the exposed vs. unexposed images.

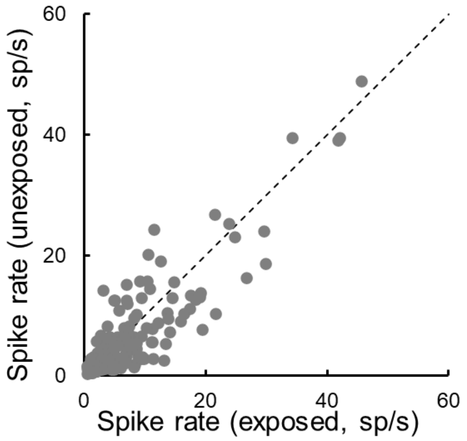

Supplementary Figure 7. Firing rates during discrimination task. Mean firing rates of individual neurons (after subtracting baseline firing) elicited by the target and test images. There are no significant changes in mean responses between the exposed (x-axis) and unexposed (y-axis) conditions (P = 0.0675, Wilcoxon sign-rank test, n = 263).

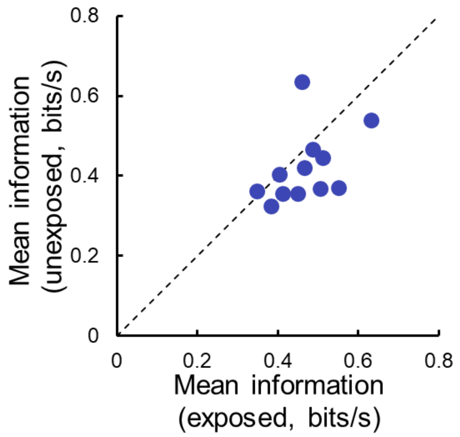

Supplementary Figure 8. Mutual information during discrimination task. Information corresponding to exposed images averaged across all the neurons in each recording session is larger than the same value for unexposed images (P = 0.042, n = 12, Wilcoxon sign-rank test).

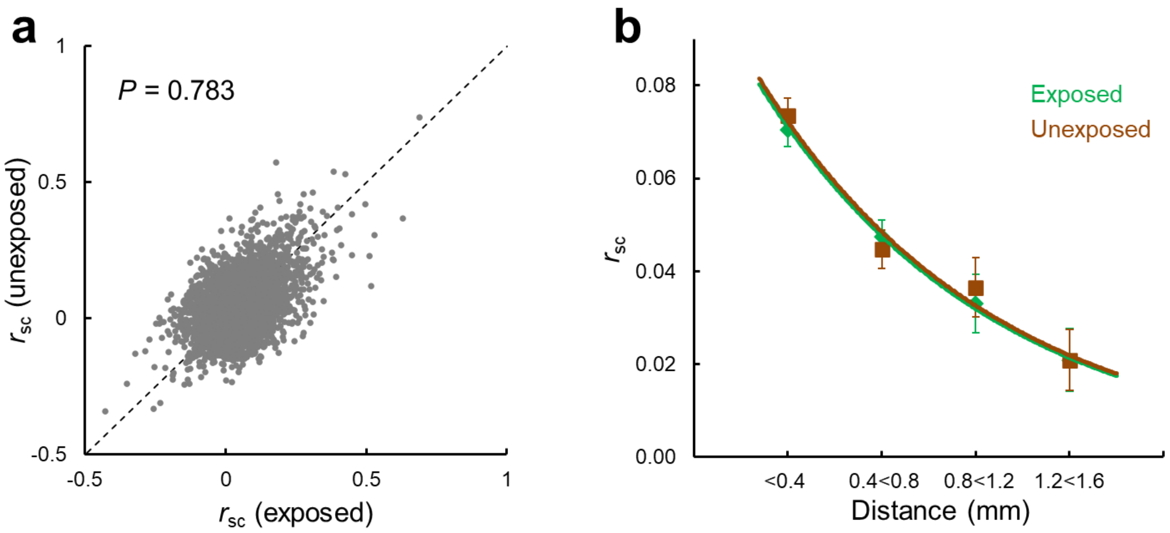

Supplementary Figure 9. Noise correlations for the exposed and unexposed images. a, Pairwise noise correlations (r_{sc} represents the correlation coefficient) are not significantly different between the exposed and unexposed stimulus conditions (P = 0.783, Wilcoxon signed-rank test). **b,** The mean Pearson correlation coefficient (r_{sc}) is calculated as a function of the cortical distance between cells. Pairs were pooled across sessions based on the distance between electrodes (n = 952, 571, 236, 144, considering only the pairs for which the cortical distance could be calculated). Error bars represent sem.

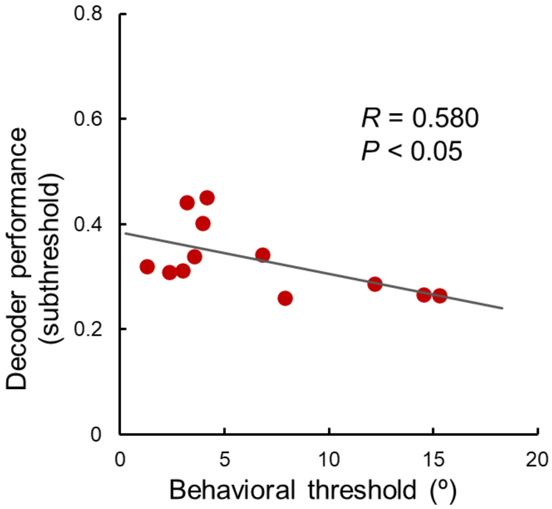

Supplementary Figure 10. Decoder performance during the exposure task is correlated with behavioral performance (discrimination threshold) during the subsequent discrimination task (R = 0.580, P = 0.048, Pearson correlation; exposed images).

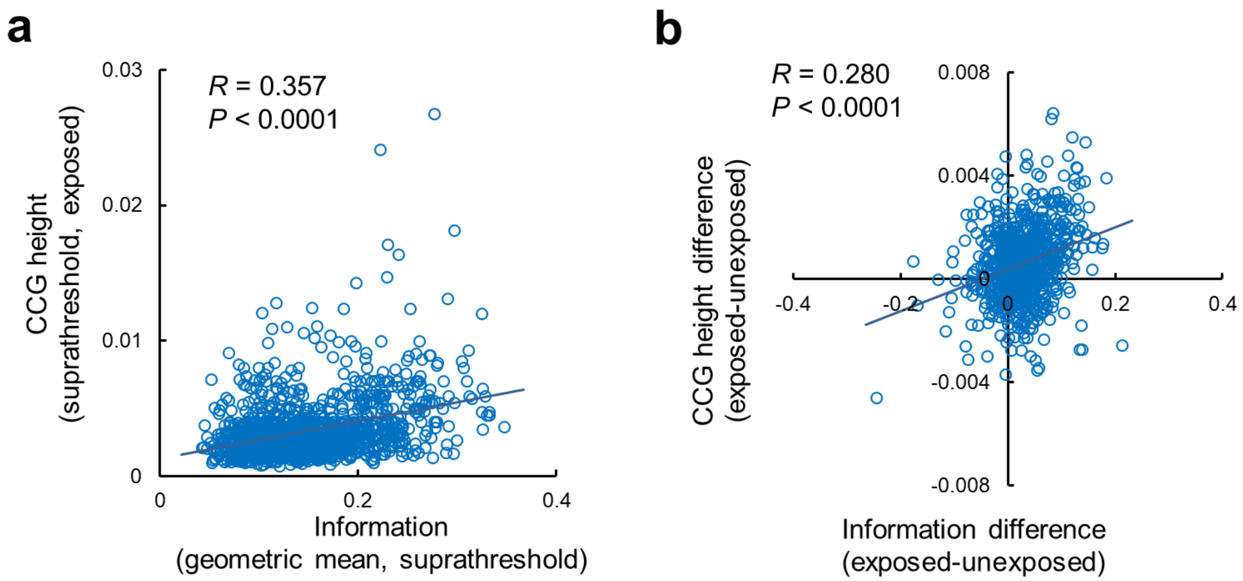

Supplementary Figure 11. Increased functional connectivity for coactive pairs of neurons. a, CCG amplitude for coactive pairs during suprathreshold presentation of previously exposed images is strongly correlated with the geometric mean of the mutual information of the same pairs of neurons ($R = 0.357$, $P = 4.5\ 10^{-41}$, Pearson correlation). **b,** The increase in CCG amplitude for exposed vs unexposed stimuli is correlated with the increase of mutual information (geometrical mean) for the same neurons ($R = 0.280$, $P = 2\ 10^{-15}$, Pearson correlation).

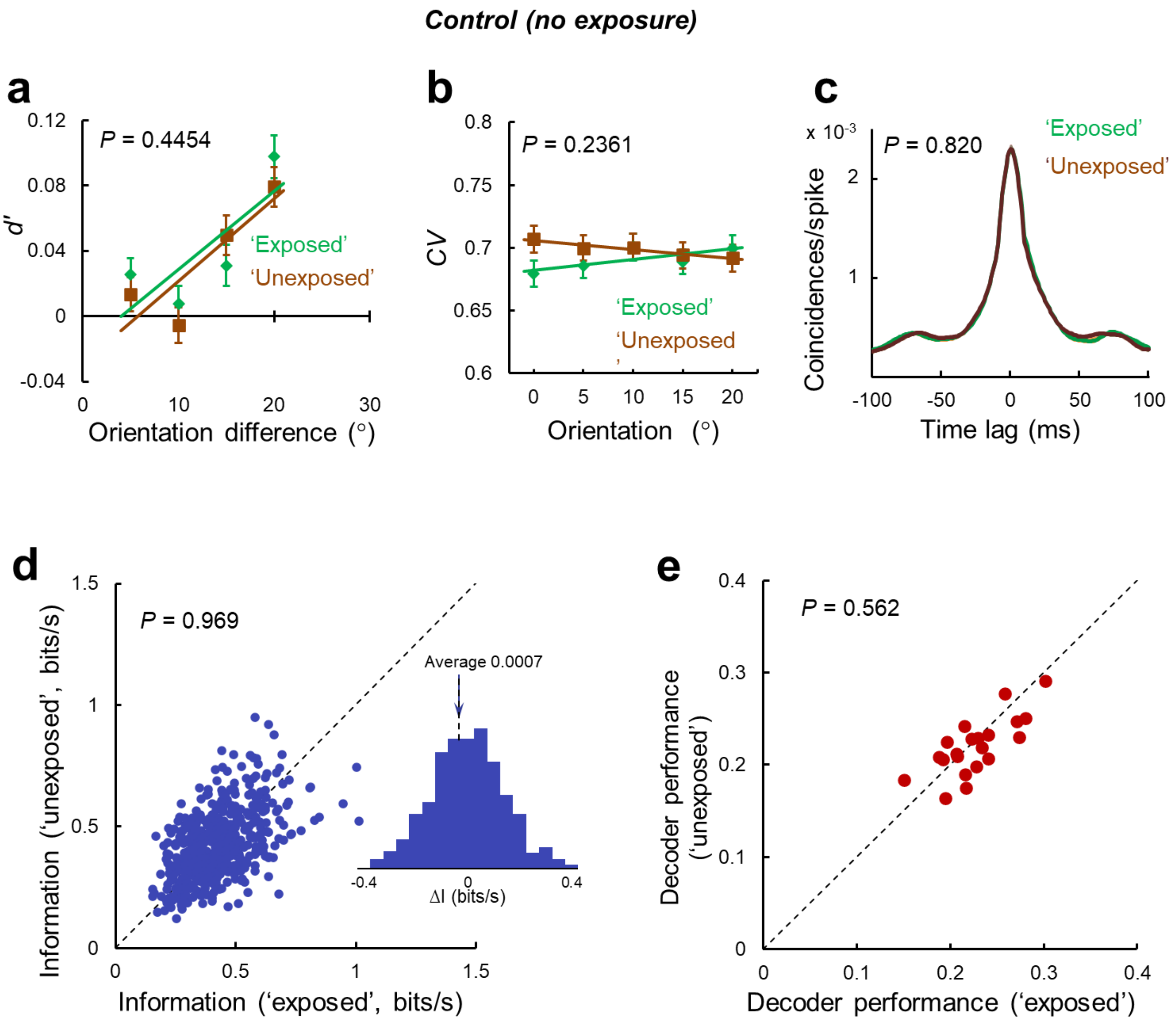

Supplementary Figure 12. Stimulus coding in the absence of exposure (45 weeks later). After 45 weeks since the original exposure experiments (**Figs. 2-5**) have ended, we performed additional controls in which the monkeys performed a fixation task in which V1 cells were exposed to flashed stimuli consisting of former 'exposed' and 'unexposed' images presented at a range of orientations. **a,** Mean neuronal sensitivity (*d'*) is not significantly different between the 'exposed' and 'unexposed' stimuli (P = 0.445, N-way ANOVA, df = 1, F = 0.58, n = 486). **b,** The mean coefficient of variation (*CV*) does not show a statistically significant difference between the two sets of images (P = 0.236, N-way ANOVA, df = 1, F = 1.4, n = 486). **c,** No significant difference in pairwise synchrony between the exposed and unexposed stimuli (P = 0.820, Wilcoxon sign-rank test). **d,** The amount of information extracted from the 'exposed' and 'unexposed' stimuli did not differ significantly (n = 486, P = 0.969, Wilcoxon sign-rank test). Each point represents mutual information for one neuron and one image pair. (inset) ΔI distribution ($I_{\text{'exposed'}} - I_{\text{'unexposed'}}$) corresponding to the data shown in panel **d**. **e,** The decoder performance calculated across image pairs is not significantly different for the 'exposed' and 'unexposed' stimuli (P = 0.562, n = 21, Wilcoxon sign-rank test). Error bars in all panels represent sem.

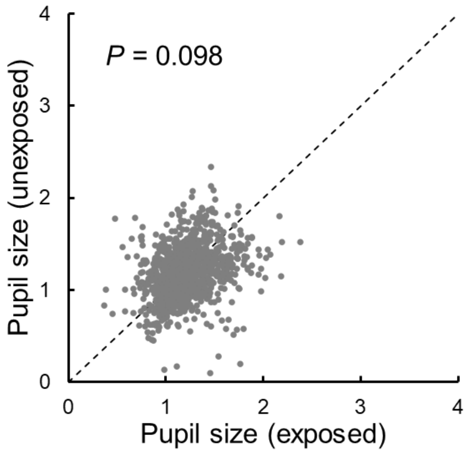

Supplementary Figure 13. Mean pupil size (represented in arbitrary units) during the discrimination task associated with exposed and unexposed images. Pupil size is not significantly different between exposed and unexposed image trials (P = 0.098, Wilcoxon sign-rank test, n = 1240 trials).
